# Cellular heterogeneity and key subsets of tissue-resident memory T cells in cervical cancer

**DOI:** 10.1038/s41698-024-00637-3

**Published:** 2024-07-16

**Authors:** Fuhao Wang, Shengqin Yue, Qingyu Huang, Tianyu Lei, Xiaohui Li, Cong Wang, Jinbo Yue, Chao Liu

**Affiliations:** 1 Department of Radiation Oncology, Peking University First Hospital, 100034 Beijing, China; 2 Department of Oncology, Renmin Hospital of Wuhan University, Wuhan, 430060 China; 3 Department of Radiation Oncology, Shandong Cancer Hospital and Institute, Shandong First Medical University and Shandong Academy of Medical Sciences, Jinan, 250117 China; 4 Department of Gynecologic Oncology, Shandong Cancer Hospital and Institute, Shandong First Medical University and Shandong Academy of Medical Sciences, Jinan, 250117 China

**Keywords:** Cervical cancer, High-throughput screening, Lymphocytes

## Abstract

Tissue-resident memory T cells (TRMs) play a critical role in cancer immunity by offering quick and effective immune responses. However, the cellular heterogeneity of TRMs and their significance in cervical cancer (CC) remain unknown. In this study, we generated and analyzed single-cell RNA sequencing data from 12,945 TRMs (*ITGAE*^*+*^ *CD3D*^*+*^) and 25,627 non-TRMs (*ITGAE*^*−*^ *CD3D*^*+*^), derived from 11 CC tissues and 5 normal cervical tissues. We found that TRMs were more immunoreactive than non-TRMs, and TRMs in CC tissues were more activated than those in normal cervical tissues. Six CD8^+^ TRM subclusters and one CD4^+^ TRM subcluster were identified. Among them, *CXCL13*^+^ CD8^+^ TRMs were more abundant in CC tissues than in normal cervical tissues, had both cytotoxic and inhibitory features, and were enriched in pathways related to defense responses to the virus. Meanwhile, *PLAC8*^+^ CD8^+^ TRMs were less abundant in CC tissues than in normal cervical tissues but had highly cytotoxic features. The signature gene set scores of both cell subclusters were positively correlated with the overall survival and progression-free survival of patients with CC following radiotherapy. Of note, the association between *HLA-E* and *NKG2A*, either alone or in a complex with *CD94*, was enriched in *CXCL13*^*+*^ CD8^+^ TRMs interacting with epithelial cells at CC tissues. The in-depth characterization of TRMs heterogeneity in the microenvironment of CC could have important implications for advancing treatment and improving the prognosis of patients with CC.

## Introduction

Cervical cancer (CC) is the fourth most common malignant tumor in women worldwide and is a leading cause of malignant tumor-related deaths^1^. Persistent infection with high-risk human papillomavirus (HPV) subtypes, particularly HPV 16 and 18, can alter cervical cells, leading to the development of pre-cancerous lesions and eventually CC^2^. Conventional cancer therapies, including surgical resection, radiotherapy, and chemotherapy, for CC are currently insufficient. Resistance to therapy causes CC recurrence and metastasis in approximately one-third of patients after treatment completion^3,4^. In recent years, immunotherapy has brought new hope to cancer patients and is especially changing the treatment landscape of advanced CC^5^. These immunotherapies include immune checkpoint inhibitors (ICIs), therapeutic vaccines, and adoptive cell transfer therapy, all of which aim to boost anti-tumor immune responses mediated by immune cells, especially T cells^6^.

Tissue-resident memory T cells (TRMs) are a special subset of T lymphocytes that reside indefinitely in tissues^7^. The presence of an infected or malignant cell initiates a T-cell-mediated immune response, following which, some T cells, which retain specific information relating to this encounter, survive. When the same infection or tumor cell re-emerges, these so called “memory T cells” can respond more quickly and effectively than their naive counterparts^8^. Among the various subsets of memory T cells, those residing in tissues and organs are called TRMs and are distinguished by the expression of the integrin CD103 (encoded by *ITGAE*)^9,10^. CD103^+^ T cells have been demonstrated to play a vital role in the immune response against solid tumors, including breast cancer^11^, lung cancer^12^, endometrial adenocarcinoma^13^, and bladder urothelial carcinoma^14^. CD49a and CD69 are important markers of TRMs but their expressions are not as widespread and consistent across different tissues as CD103. CD69 is a T cell activation marker that can be expressed in other T cell subsets beyond TRMs, including activated effector T cells[15, 16]. CD49a expression might be used to distinguish TRM subsets with different effector functions and putative roles in immunopathology. In skin from patients with vitiligo, CD8^+^CD49a^+^ TRMs that constitutively express perforin and GZMB accumulates in the epidermis and dermis. Conversely, CD8^+^CD49a^-^ TRMs from psoriasis lesions predominantly generate IL-17 responses that promote local inflammation in this skin disease[17]. Therefore, we chose CD103 as the most widely recognized and strongly associated single marker to ensure the reliability of our research and the clarity of the results in this study. Currently, researchers are seeking ways to activate and enhance the efficacy of TRM responses as a potential strategy for cancer immunotherapy. Moreover, CD8^+^ TRMs express high levels of immune checkpoint molecules and are thought to be early responders to ICIs treatment^15^. In addition, cancer vaccines, which prime TRMs to specifically target cancer cells, are being developed and have shown objective efficacy in inhibiting the growth of melanoma tumors in mucosal tissues^16,17^. Furthermore, a novel adoptive T cell therapeutic strategy, which aims to collect TRMs from resected tumor tissues, also brings new challenges and opportunities to the field of cancer therapy^18^. Thus, the use of TRMs in cancer immunotherapy is a field of active research, and continued studies are necessary to determine their feasibility as an effective cancer treatment.

In recent times, the field of cancer research has been advanced by the emergence of single-cell RNA sequencing (scRNA-seq) technology^19^. Compared with bulk RNA sequencing, scRNA-seq enables the study of individual cells, providing researchers with a measure of cellular heterogeneity within complex tissues or cell populations. scRNA-seq can identify rare cell types or states, which may otherwise be missed in a bulk RNA analysis^20^. However, there are currently no scRNA-seq studies deciphering the TRMs landscape of CC. A thorough dissection of the cellular heterogeneity and molecular characteristics of TRMs in the tumor microenvironment (TME) of CC at the single-cell level is crucial for developing novel immunotherapies and improving patient prognosis.

In our study, we analyzed scRNA-seq data from 38,572 T cells, derived from 11 CC and 5 normal cervical tissues. Our data revealed that TRMs demonstrated significantly higher levels of immunoreactivity than non-TRMs; and TRMs within CC tissues were more activated than those in normal cervical tissues. Furthermore, we identified two subclusters of CD8^+^ TRMs, expressing either *CXCL13* or *PLAC8*, which had important roles in the TME of CC but differed in their abundance, molecular characteristics, cell interactions, and prognostic significance. We believe that these findings will help guide the development of diagnostic and/or therapeutic approaches to improve the prognosis of patients with CC.

## Results

### Single-cell transcriptomes revealed that TRMs are more immunoreactive than non-TRMs

**Fig. 1:**
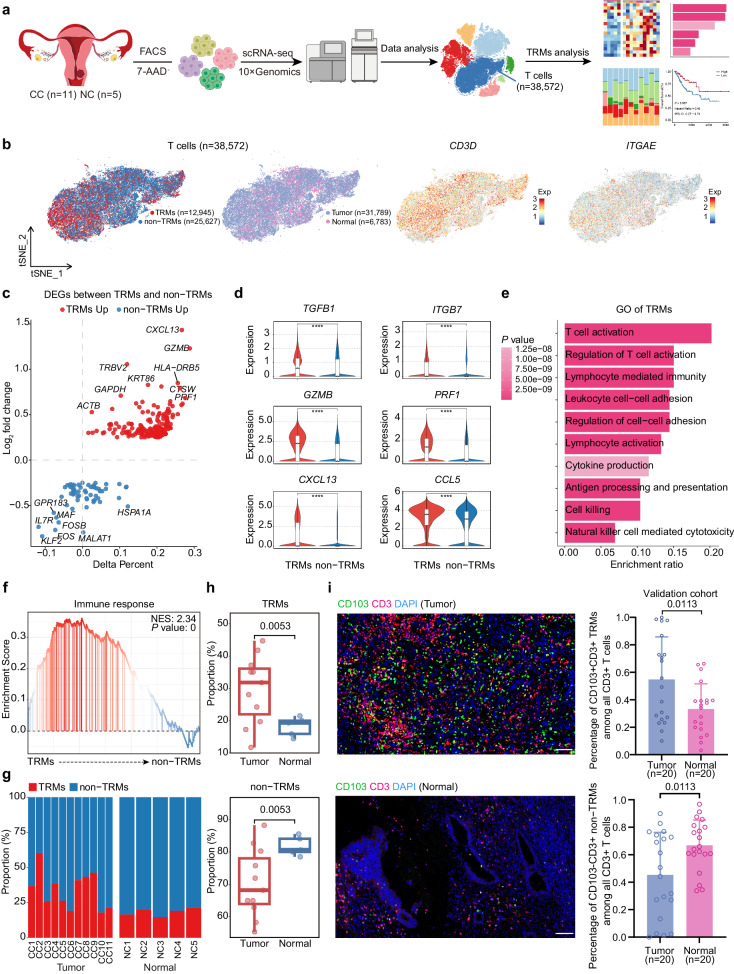
TRMs are more immunoreactive than non-TRMs. **a** Overview of the experimental workflow. **b** t-Distributed Stochastic Neighbor Embedding (tSNE) plots showing subcluster, sample origin, and marker gene expression. The color key shows the gradient of normalized expression. **c** Volcano plot displaying differentially expressed genes (DEGs) between TRMs (red) and non-TRMs (blue). **d** Violin plots illustrating the expression of the indicated genes in TRMs (red) and non-TRMs (blue). *p < 0.05; **p < 0.01; ***p < 0.001; ****p < 0.0001 (two-sided Wilcoxon test). **e** Gene ontology (GO) term analysis in TRMs. The intensity of color indicates *p*-value. **f** Gene Set Enrichment Analysis (GSEA) showing that TRMs were enriched in gene sets associated with the immune response. NES, normalized enrichment score. **g** Bar plots depicting the proportions of TRMs (red) and non-TRMs (blue) in each sample. **h** Box plots illustrating the proportions of cell subclusters in normal cervix and cervical cancer (CC) tissues. **i** Representative immunofluorescent labeling of CD103 (green), CD3 (red), and 4,6-diamidino-2-phenylindole (DAPI, blue) in sections from CC (up) and normal cervical tissues (down). Box plots showing the percentage of CD103^+^ CD3^+^ TRMs and CD103^-^ CD3^+^ non-TRMs in CC and normal cervical tissues based on immunofluorescent labeling results. Scale bar in figure, 80 μm. The *p*-values were generated using the Wilcoxon test.

We obtained scRNA-seq data from 38,572 T cells from 11 CC and 5 normal cervical samples (Fig. 1a). T cells were classified as TRMs (12,945 cells; *ITGAE*^+^ *CD3D*^+^) or non-TRMs (25,627 cells; *ITGAE*^−^ *CD3D*^+^) on the basis of *ITGAE* expression (Fig. 1b), consistent with TRMs described in previous researches^21,22^. In addition, we compared the expression of *ITGAE* in TRMs, non-TRMs, and T cells in peripheral blood mononuclear cells (PBMC), further validating the specificity of *ITGAE* for defining TRMs (Supplementary Fig. 1d). Differentially expressed genes (DEGs) analysis revealed that TRMs expressed higher levels of genes associated with tissue residence (*TGFB1* and *ITGB7*), cytotoxicity (*GZMB* and *PRF1*), and chemokine production (*CXCL13* and *CCL5*) than non-TRMs (Fig. 1c, d). The gene ontology (GO) term analysis showed that DEGs related to the cell adhesion pathway were enriched in TRMs than non-TRMs, which is associated with the retention of T cells in tissues. In addition, pathways associated with T cell activation, lymphocyte-mediated immunity, and antigen processing and presentation were also enriched in TRMs than non-TRMs (Fig. 1e, f and Supplementary Fig. 1c). Moreover, TRMs were more abundant in CC tissues than in normal cervical tissues (*p* = 0.0053; Fig. 1g, h). Subsequently, we confirmed that the infiltration degree of TRMs in CC was higher than that in normal cervical tissues by performing immunofluorescent labeling on formalin-fixed and paraffin-embedded (FFPE) samples of CC and normal cervical tissues (*p* = 0.0113; Fig. 1i). These findings indicate that TRMs are more abundant and immunoreactive than non-TRMs in the TME of CC.

### Single-cell transcriptome analysis revealed that TRMs are more activated in CC tissues than in normal cervical tissues

**Fig. 2:**
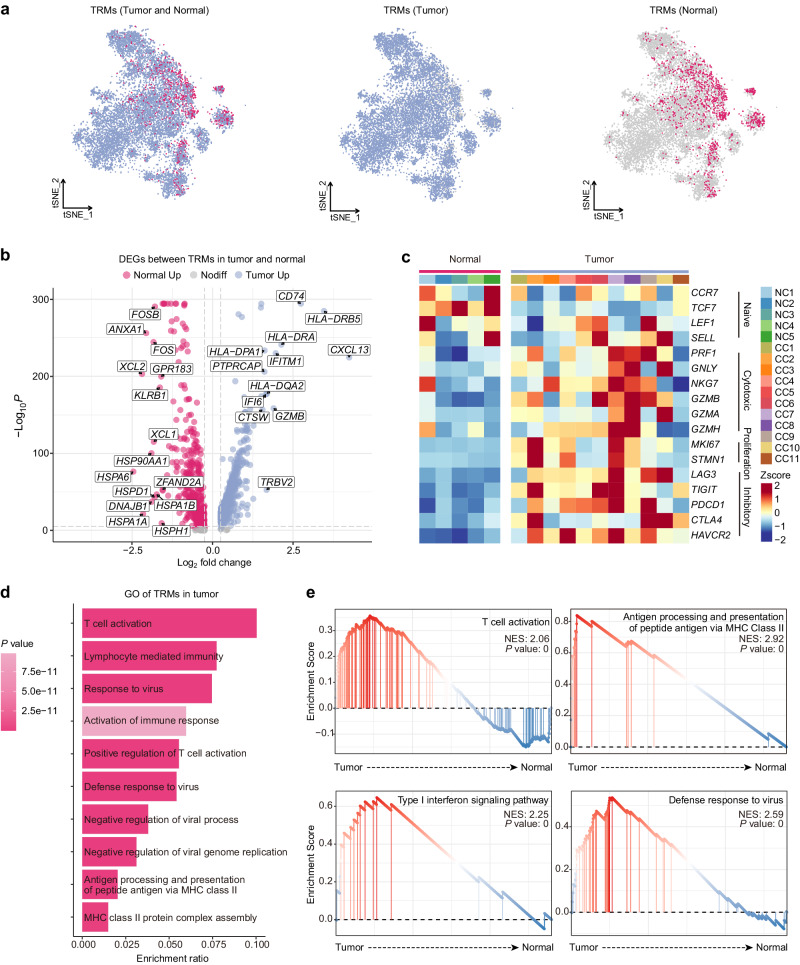
Characteristics of TRMs in the TME of CC. **a** tSNE plots showing distribution of TRMs from CC tissues (blue) and those from normal cervical tissues (pink). **b** Volcano plot showing DEGs between TRMs from CC tissues and those from normal cervical tissues. **c** Heatmap showing the expression of functional signature genes, whereby color intensity indicates average gene expression. **d** GO term analysis in TRMs from CC tissues. The color intensity indicates *p*-value. **e** GSEA showing differences in the activation of various immune cell pathways in TRMs from CC tissues and in those from normal cervical tissues.

Based on the higher infiltration of TRMs in CC tissues than in normal ones, we next explored the transcriptomes of TRMs in CC tissues and in normal cervical tissues (Fig. 2a). We first identified DEGs between TRMs from CC tissues and those from normal cervical tissues (Fig. 2b). Functional gene set analysis revealed that TRMs from CC tissues not only expressed more genes related to cytotoxicity and proliferation, but also expressed higher levels of genes encoding immune checkpoint molecules than TRMs from normal cervical tissues. By contrast, the TRMs from normal cervical tissues had a more naïve-like T cell signature (Fig. 2c). The GO term analysis of DEGs showed that TRMs in CC tissues were enriched in pathways related to T cell activation, antigen processing and presentation of peptide antigen via MHC class II, and lymphocyte-mediated immunity. They were also enriched in pathways associated with defense response to virus (Fig. 2d and Supplementary Fig. 2). The gene set enrichment analysis (GSEA) results confirmed the enrichment of gene sets related to T cell activation, antigen processing and presentation of peptide antigen via MHC class II, as well as the response to type I interferon and defense response to virus (Fig. 2e). These results suggest that TRMs residing in the TME of CC are more activated than those localized to normal cervical tissues; moreover, TRMs may play a crucial role in the anti-viral immune response in the cervix.

### Single-cell transcriptome analysis of TRMs heterogeneity

**Fig. 3:**
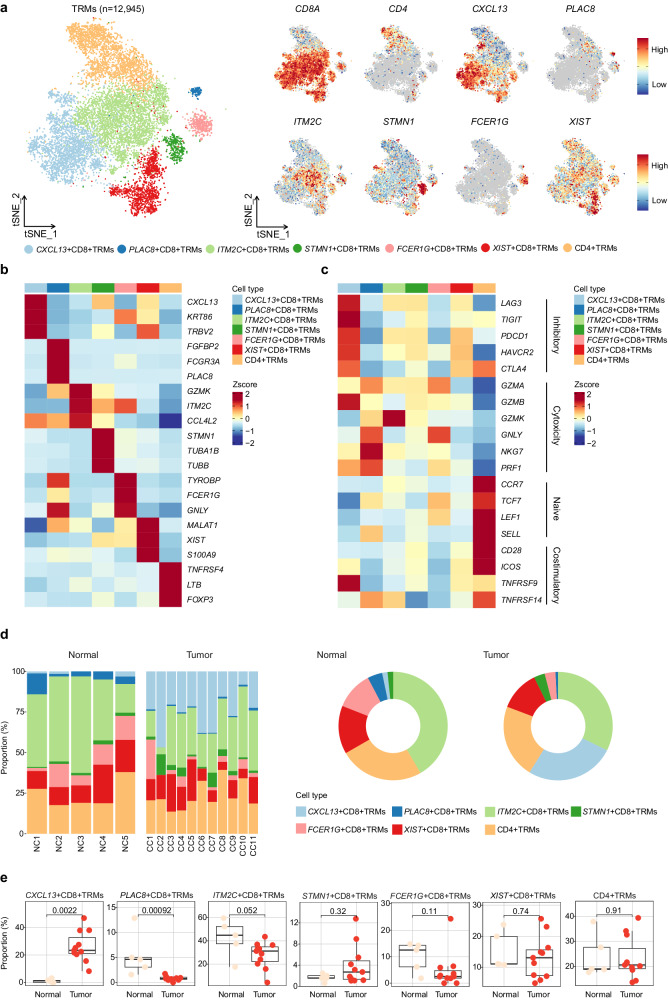
Single-cell transcriptome profiles of 12,945 TRMs. **a** tSNE plots showing the TRM subclusters and the expression of marker genes; the color key shows the gradient of normalized expression. Heatmaps displaying the relative expression of the top 3 DEGs (**b**) and functional signature genes (**c**) in the seven TRM subclusters; the color intensity reflects the average level of gene expression. **d** The proportions of the seven TRM subclusters in each tissue and type (CC tissues vs. normal cervical tissues), colored by cell types. **e** Box plots illustrating the proportions of TRM subclusters in normal cervical (pink) and CC (red) tissues. The *p*-values were calculated using the paired Wilcoxon test.

To further dissect the molecular heterogeneity of TRMs, we extracted data from all the TRMs for use in a sub-clustering analysis. We obtained seven TRM subclusters: six CD8^+^ TRM subclusters, differentiated by *CXCL13*, *PLAC8*, *ITM2C, STMN1, FCER1G*, or *XIST* expression; and one CD4^+^ TRM cluster (Fig. 3a). These TRM subclusters exhibited widely divergent gene expression profiles (Fig. 3b and Supplementary Fig. 3a). Among them, *CXCL13*^+^ CD8^+^ TRMs demonstrated elevated expression of genes encoding inhibitory molecules (*LAG3*, *TIGIT*, *PDCD1*, *HAVCR2*, and *CTLA4*) and had a higher inhibitory score than other TRM subclusters. Additionally, *CXCL13*^+^ CD8^+^ TRMs also expressed genes encoding cytotoxic molecules (*GZMB* and *PRF1*; Fig. 3c and Supplementary Fig. 3b). In addition, *PLAC8*^+^ CD8^+^ TRMs demonstrated elevated expression of genes encoding cytotoxic molecules (*GZMA*, *GNLY*, *NKG7*, and *PRF1*; Fig. 3c) and had a higher naïve score than other CD8^+^ TRM subclusters (Supplementary Fig. 3b). The relative abundances of the seven major TRM subclusters in the CC tissues and normal cervical tissues were shown in Fig. 3d. Further comparisons revealed that the proportion of infiltrating *CXCL13*^+^ CD8^+^ TRMs was significantly higher in CC tissues than in normal cervical tissues (*p* = 0.0022), whereas the proportion of infiltrating *PLAC8*^+^ CD8^+^ TRMs was significantly lower in CC tissues than in normal cervical tissues (*p* = 0.00092; Fig. 3e).

### *CXCL13*^+^ CD8^+^ TRMs are enriched in CC tissues and express transcripts related to antigen processing and presentation and defense responses to the virus

**Fig. 4:**
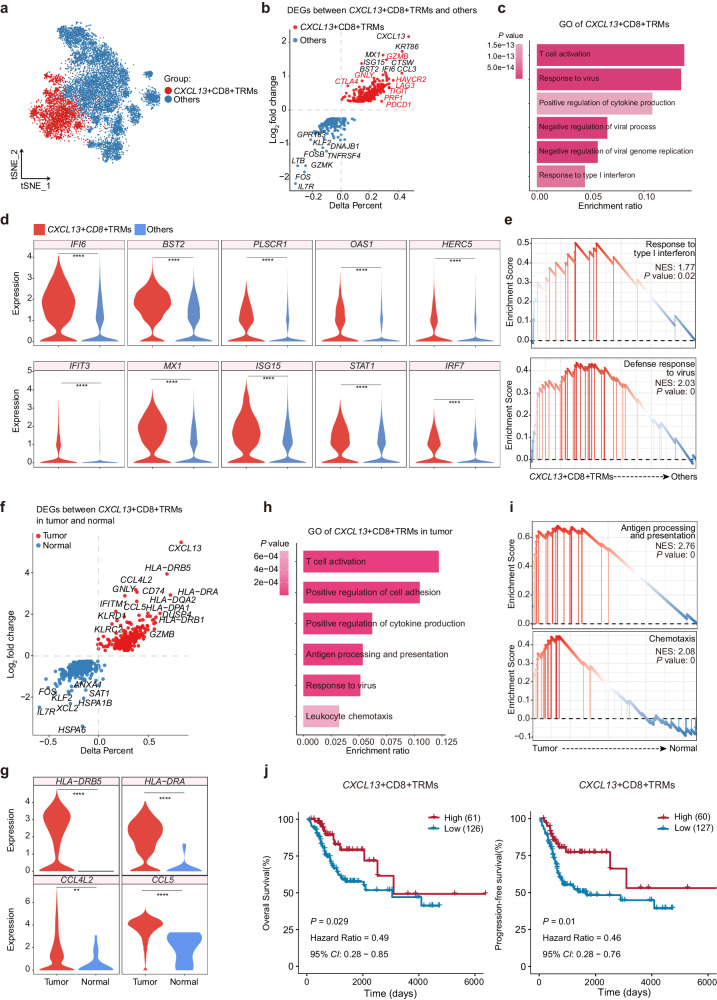
Characteristics of *CXCL13*^+^ CD8^+^ TRMs. **a** tSNE plot showing the distribution of *CXCL13*^+^ CD8^+^ TRMs (red) relative to that of the other TRM subclusters (blue). **b** Volcano plot depicting DEGs between *CXCL13*^+^ CD8^+^ TRMs (red) and the other TRM subclusters (blue). **c** GO term analysis of *CXCL13*^+^ CD8^+^ TRMs. The color intensity indicates *p*-value magnitude. **d** Violin plots illustrating the expression of the indicated genes in *CXCL13*^+^ CD8^+^ TRMs and the other TRM subclusters. *p < 0.05; **p < 0.01; ***p < 0.001; ****p < 0.0001 (two-sided Wilcoxon test). **e** GSEA showing differences in pathway activity between *CXCL13*^+^ CD8^+^ TRMs and the other TRM subclusters. **f** Volcano plot depicting the DEGs between *CXCL13*^+^ CD8^+^ TRMs from CC tissues (red) and those from normal cervical tissues (blue). **g** Violin plots displaying the expression of the indicated genes in *CXCL13*^+^ CD8^+^ TRMs from CC tissues and in those from normal cervical tissues. *p < 0.05; **p < 0.01; ***p < 0.001; ****p < 0.0001 (two-sided Wilcoxon test). **h** GO term analysis of *CXCL13*^+^ CD8^+^ TRMs from CC tissues; color intensity indicates *p*-value magnitude. **i** GSEA results showing differences in the pathway activities of *CXCL13*^+^ CD8^+^ TRMs from CC tissues and those from normal cervical tissues. **j** Kaplan–Meier survival analysis of patients with CC following radiotherapy from TCGA database, stratified based on high or low signature gene set score of *CXCL13*^+^ CD8^+^ TRMs. The *p*-values were calculated using the two-sided log-rank test.

To determine the unique molecular signature of *CXCL13*^+^ CD8^+^ TRMs, we identified DEGs between *CXCL13*^+^ CD8^+^ TRMs and the other TRM subclusters. The result exhibited that *CXCL13*^+^ CD8^+^ TRMs showed high expression of cytotoxic genes (*GZMB*, *GNLY*, and *PRF1*) and inhibitory genes (*HAVCR2*, *TIGIT*, *LAG3*, *CTLA4*, and *PDCD1*) (Fig. 4a, b). The GO term analysis revealed that *CXCL13*^+^ CD8^+^ TRMs were enriched in pathways associated with T cell activation, cytokine production, as well as response to virus and response to type I interferon (Fig. 4c). Moreover, *CXCL13*^+^ CD8^+^ TRMs expressed higher level of genes related to defense response to virus, including *IFI6, IFIT3, BST2, MX1, PLSCR1, ISG15, OAS1, STAT1, HERC5*, and *IRF7*, than the other TRM subclusters (Fig. 4d). The GSEA results also confirmed that *CXCL13*^+^ CD8^+^ TRMs were enriched in gene sets associated with defense response to virus and response to type I interferon (Fig. 4e and Supplementary Fig. 4). These findings suggest that *CXCL13*^+^ CD8^+^ TRMs may exert anti-viral effects upon stimulation by type I interferon.

Because *CXCL13*^+^ CD8^+^ TRMs were predominantly found in CC tissues, we explored the differences in the signatures of *CXCL13*^+^ CD8^+^ TRMs from CC tissues and normal cervical tissues. DEGs analysis revealed that MHC class II molecule genes (*HLA-DRB5* and *HLA-DRA*) and chemokine-related genes (*CCL4L2* and *CCL5*) were highly expressed in *CXCL13*^+^ CD8^+^ TRMs from CC tissues (Fig. 4f, g). The GO term analysis revealed that *CXCL13*^+^ CD8^+^ TRMs from CC tissues were enriched in pathways related to antigen processing and presentation, cell adhesion, and chemotaxis (Fig. 4h); this was confirmed by the results of the GSEA (Fig. 4i). We therefore next evaluated the relationship between *CXCL13*^+^ CD8^+^ TRM signature and the survival outcomes of patients with CC following radiotherapy using bulk RNA-seq data obtained from The Cancer Genome Atlas (TCGA). We found that the signature gene set score of *CXCL13*^+^ CD8^+^ TRMs was positively correlated with the overall survival (OS) and progression-free survival (PFS) of patients with CC following radiotherapy (Fig. 4j).

In summary, these findings indicate that *CXCL13*^+^ CD8^+^ TRMs exhibit cytotoxic and inhibitory characteristics, enriched in pathways associated with defense response to virus and the type I interferon response than the other TRM subclusters, and are associated with the improved outcome of patients with CC following radiotherapy.

### *PLAC8*^+^ CD8^+^ TRMs are less enriched in CC tissues than in normal cervical tissues but exhibit highly cytotoxic characteristics

**Fig. 5:**
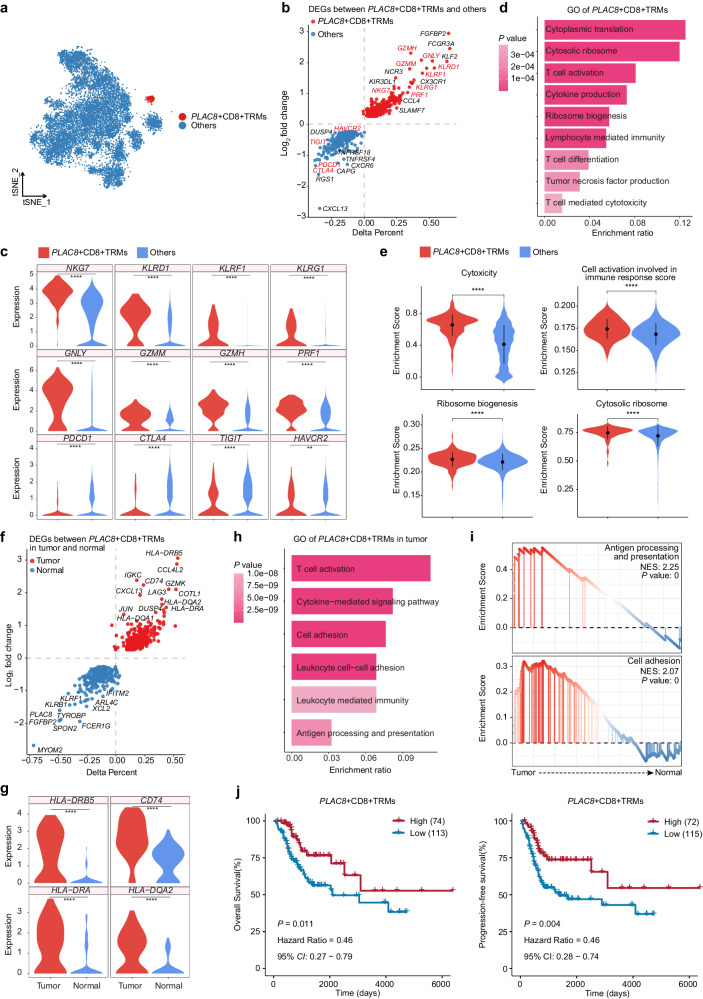
Characteristics of *PLAC8*^+^ CD8^+^ TRMs. **a** tSNE plot showing the distribution of *PLAC8*^+^ CD8^+^ TRMs (red) relative to that of the other TRM subclusters (blue). **b** Volcano plot showing DEGs between *PLAC8*^+^ CD8^+^ TRMs (red) and the other TRM subclusters (blue). **c** Violin plots displaying the expression of the indicated genes between *PLAC8*^+^ CD8^+^ TRMs and the other TRM subclusters. *p < 0.05; **p < 0.01; ***p < 0.001; ****p < 0.0001 (two-sided Wilcoxon test). **d** GO term analysis of *PLAC8*^+^ CD8^+^ TRMs; the color intensity indicates *p*^-^value magnitude. **e** Violin plots showing differences in pathway activity between *PLAC8*^+^ CD8^+^ TRMs (red) and the other TRM subclusters (blue). **f** Volcano plot showing DEGs in *PLAC8*^+^ CD8^+^ TRMs between CC tissues (red) and normal cervical tissues (blue). **g** Violin plots illustrating the expression of indicated genes in *PLAC8*^+^ CD8^+^ TRMs between CC tissues and normal cervical tissues. *p < 0.05; **p < 0.01; ***p < 0.001; ****p < 0.0001 (two-sided Wilcoxon test). **h** GO term analysis in *PLAC8*^+^ CD8^+^ TRMs of CC tissues. The intensity of color indicates *p*-values. **i** GSEA showing differences in the pathway activities of *PLAC8*^+^ CD8^+^ TRMs from CC tissues and those from normal cervical tissues. **j** Kaplan–Meier survival analysis of patients with CC following radiotherapy from TCGA database, stratified based on high or low signature gene set score of *PLAC8*^+^ CD8^+^ TRMs. The *p*-values were calculated using a two-sided log-rank test.

We next dissected the molecular signature of *PLAC8*^+^ CD8^+^ TRMs, the subcluster which was less abundant in CC tissues than in normal cervical tissues (Fig. 5a). DEGs analysis exhibited that *PLAC8*^+^ CD8^+^ TRMs showed high expression of cytotoxic genes (*GZMH*, *GNLY*, *GZMM*, *KLRD1*, *KLRF1*, *KLRG1*, *NKG7*, and *PRF1*) and low expression of genes encoding inhibitory molecules (*PDCD1*, *CTLA4, TIGIT*, and *HAVCR2*) (Fig. 5b, c), exhibiting similar characteristics to cytotoxic T cells. Further GO term analysis revealed that *PLAC8*^+^ CD8^+^ TRMs were enriched in pathways associated with T-cell-mediated immune effector processes, and also enriched cytoplasmic translation and ribosome biogenesis pathways (Fig. 5d, e).

In addition, we explored signature differences between *PLAC8*^+^ CD8^+^ TRMs from CC tissues and those from normal cervical tissues. DEGs analysis revealed that *HLA-DRB5*, *CD74, HLA-DRA*, and *HLA-DQA2* were more highly expressed in *PLAC8*^+^ CD8^+^ TRMs from CC tissues than in those from normal cervical tissues (Fig. 5f, g). The GO term analysis demonstrated that *PLAC8*^+^ CD8^+^ TRMs from CC tissues were enriched with immune response-related pathways, including T cell activation, cytokine-mediated signaling pathway, antigen processing and presentation, and cell adhesion (Fig. 5h and Supplementary Fig. 5). The GSEA confirmed that *PLAC8*^+^ CD8^+^ TRMs were enriched in gene sets associated with antigen processing and presentation, as well as cell adhesion (Fig. 5i). Moreover, survival analysis revealed a positive correlation between the signature gene set score of *PLAC8*^+^ CD8^+^ TRMs and the OS and PFS of patients with CC following radiotherapy (Fig. 5j). These findings suggest that although *PLAC8*^+^ CD8^+^ TRMs were not abundant in the TME of CC, they were a highly cytotoxic, readily activated, clinically important TRM subset.

### *CXCL13*^*+*^ CD8^+^ TRMs and *PLAC8*^*+*^ CD8^+^ TRMs interact with epithelial cells in the TME of CC

**Fig. 6:**
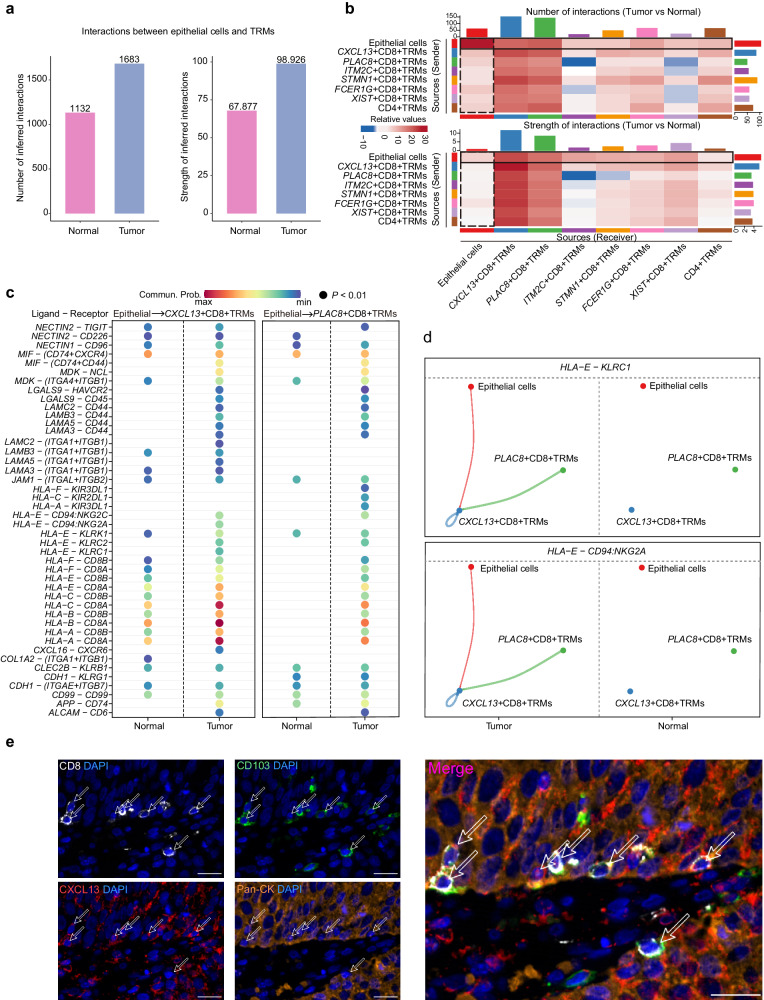
Crosstalk between epithelial cells and TRMs. **a** Bar graphs displaying the number and strength of interactions between epithelial cells and TRMs in normal cervical tissues and CC tissues, respectively. **b** CellChat analysis of scRNA-seq data to identify cell-cell communications between epithelial cells and TRM subclusters in CC tissues. The color intensity indicates the number (top panel) and strength (bottom panel) of the cell-cell interactions. **c** Bubble plots showing differences in ligand-receptor pairs involved in interactions between epithelial cell-*CXCL13*^+^ CD8^+^ TRM and epithelial cell-*PLAC8*^+^ CD8^+^ TRM in normal cervical tissues and CC tissues. Bubble size and color intensity indicate *p*-value magnitude and communication probability, respectively. **d** Circle plots showing the indicated ligand-receptor pairs involved in interactions between epithelial cells and either *CXCL13*^+^ CD8^+^ TRMs, or *PLAC8*^+^ CD8^+^ TRMs in CC tissues (left) and normal cervical tissues (right); color-coded by cell type. **e** Representative immunofluorescent labeling of Pan-CK (yellow), CXCL13 (red), CD103 (green), CD8 (white), and DAPI (blue) to identify epithelial cells, *CXCL13*^+^ CD8^+^ TRMs, and their spatial relationship in CC sections. Scale bar in figure, 20 μm.

To further investigate the interaction between epithelial cells and TRMs, we used CellChat to interrogate the scRNA-seq data and gather evidence of cell-cell communication through the analysis of ligand-receptor complexes. We found that the interactions between epithelial cells and TRMs were more robust in CC tissues than in normal cervical tissues, both in terms of their number and strength (Fig. 6a). Specifically, epithelial cells, as senders, showed more active interactions with *CXCL13*^*+*^ CD8^+^ TRMs and *PLAC8*^+^ CD8^+^ TRMs than the other TRM subclusters in CC tissues, both in terms of number and strength (Fig. 6b and Supplementary Fig. 6a).

Next, we explored the specific ligand-receptor pairs which participated in the interactions between epithelial cells and either *CXCL13*^*+*^ CD8^+^ TRMs or *PLAC8*^*+*^ CD8^+^ TRMs both in CC tissues and in normal cervical tissues (Fig. 6c). We found that MHC class I molecules-related pairs (*HLA-A/B/C/E/F - CD8A/ CD8B*), T cell proliferation and activation-related pairs (*MIF-(CD74+CD44)(CD74*+*CD44)* and *MDK-NCL*) were more active both in epithelial cell*-CXCL13*^*+*^ CD8^+^ TRM and epithelial cell*-PLAC8*^*+*^ CD8^+^ TRM at CC tissues than normal cervical tissues (Fig. 6c and Supplementary Fig. 6b). This result suggested that tumor antigens may be presented to TRMs via MHC class I molecules in the TME of CC, leading to the rapid activation of this T cell subset and the induction of anti-tumor immune responses. In addition, *HLA-E - KLRC1* and *HLA-E - CD94:NKG2A* pairs were enriched in epithelial cell*-CXCL13*^*+*^ CD8^+^ TRM at CC tissues, rather than in epithelial cell-*PLAC8*^*+*^ CD8^+^ TRM (Fig. 6c, d). Of note, both of these interactions are associated with T cell inhibition and increased cancer growth^23,24^. We confirmed the presence and spatial relationship of epithelial cells and *CXCL13*^*+*^ CD8^+^ TRMs within the TME of CC by multiplex immunofluorescent staining by labeling for pan-cytokeratin (Pan-CK), CXCL13, CD103, and CD8. The proximity observed between epithelial cells and *CXCL13*^*+*^ CD8^+^ TRMs on tissue sections supports their potential cellular interactions (Fig. 6e).

In summary, we detected active interactions between epithelial cells and either *CXCL13*^*+*^ CD8^+^ TRMs, or *PLAC8*^*+*^ CD8^+^ TRMs in the TME of CC. Moreover, the immunosuppressive *HLA-E - KLRC1* and *HLA-E - CD94:NKG2A* pairs were enriched in *CXCL13*^*+*^ CD8^+^ TRMs interacting with epithelial cells at CC tissues.

## Discussion

TRMs play a vital role in the immune response against tumors^9^. Previous studies have dissected the cellular heterogeneity and molecular profiles of conventional T cells within the TME of CC^25–27^; however, the same in-depth characterization of TRMs has not been performed in the CC setting. Thus, in this study, we employed scRNA-seq to investigate the population characteristics of TRMs in CC. Our findings reveal that TRMs readily infiltrate CC tissues and exhibit high levels of immune activity. Furthermore, our data indicate that TRMs generate effective anti-tumor immune responses, recruit other immune cells, and respond to viral stimuli.

We initially observed that TRMs were more immunoreactive than non-TRMs, as evidenced by the high expression of immune-related genes, including those associated with cytotoxicity and chemokine production. This suggested that TRMs may kill tumor cells and recruit other immune cells to elicit effective anti-tumor immune responses; these properties of TRMs have also been reported in the context of breast cancer^28^ and lung cancer^22^. Additionally, we observe a higher level of TRM infiltration into CC tissue than normal cervical tissues, which aligns with the findings of a prior study^29^. The factors contributing to the development of tumor-infiltrating TRMs are not yet fully understood^9^. However, it is generally believed that the upregulation of migratory molecules, such as the chemokine receptors CXCR3 and CXCR6, or tissue-specific homing molecules, may play a crucial role in facilitating the recruitment of memory precursor-like cells into tumors^30^. We found that TRMs were enriched in genes associated with antigen processing and presentation, indicating that the activation of TRMs in the TME of CC may rely on MHC class II-mediated antigen presentation. Notably, the enrichment of gene sets associated with the response to type I interferon and defense response to virus in TRMs from CC tissues suggests that TRMs might exert anti-viral effects upon stimulation with type I interferon. This finding is especially relevant in the context of CC, where HPV infection plays a crucial role in tumor development^31,32^.

TRMs exhibited an activated and functional phenotype in CC tissues, expressing genes encoding cytotoxic and immune checkpoint molecules, as well as those implicated in cell proliferation. Previous studies in melanoma and lung cancer^33,34^ have shown that TRMs enriched in immune-checkpoint-related genes are especially sensitive to checkpoint inhibition therapy. TRMs play an important role in cancer immunotherapy in some solid tumors. For instance, in head and neck squamous cell carcinoma patients, neoadjuvant immunotherapy resulted in the preferential expansion of TRMs^15^. Encouragingly, blockade of PD-1 and/or CTLA-4 effectively restored TRMs proliferation and enhanced their cytokine production^35^. Moreover, anti-PD-1 therapy promoted TRMs proliferation in melanoma patients, which was associated with improved survival^36^.

We identified two significant subgroups of TRMs in CC. *CXCL13*^*+*^ CD8^+^ TRMs were more abundant in CC tissues compared to normal cervical tissues and had both cytotoxic (*GZMB*, *GNLY*, and *PRF1*) and inhibitory (*HAVCR2*, *TIGIT*, *LAG3*, *CTLA4*, and *PDCD1*) characteristics. In vitro experiments have demonstrated that blocking immune checkpoints, such as PD-1 and TIM-3, enhances the proliferation of CD8^+^ TRMs and improves their cytokine-mediated killing capacity^37,38^. These findings suggest that *CXCL13*^*+*^ CD8^+^ TRMs, which express immune-checkpoint molecules, could be effectively targeted by ICIs therapy. Interestingly, *CXCL13*^*+*^ CD8^+^ TRMs were more enriched in pathways associated with defense response to virus and the type I interferon response than the other TRM subclusters. Previous studies have shown the significance of TRMs in the immune against HPV infection, as they can recognize HPV-infected cells and contribute to viral clearance^39^. The unique profile of *CXCL13*^*+*^ CD8^+^ TRMs suggests that they may exert anti-HPV effects.

By contrast, *PLAC8*^*+*^ CD8^+^ TRMs was less abundant in CC tissues compared to normal cervical tissues and exhibited a higher naïve score than other CD8^+^ TRM subclusters. We hypothesize that upon residency within CC tissues, *PLAC8*^*+*^ CD8^+^ TRMs undergo gradual differentiation into other TRM subgroups in response to persistent stimulation by tumor-related antigens. *PLAC8*^*+*^ CD8^+^ TRMs exhibited elevated expression of cytotoxic genes and reduced expression of genes encoding inhibitory molecules. Moreover, they were enriched in pathways associated with T-cell-mediated immune effector processes. These findings indicate that *PLAC8*^*+*^ CD8^+^ TRMs are important in immune function. And the upregulation of genes associated with cytoplasmic translation and ribosome biogenesis suggests that *PLAC8*^*+*^ CD8^+^ TRMs have a high protein synthesis capacity. To recognize and kill target cells, including tumor cells, cytotoxic T cells need to rapidly produce and release cytotoxic proteins, such as granzymes and perforin^40^. This increased demand for protein following T cell activation requires the up-regulation of ribosome biogenesis^41^. Importantly, the frequencies of *CXCL13*^*+*^ CD8^+^ TRMs and *PLAC8*^*+*^ CD8^+^ TRMs exhibited a positive correlation with the prolonged survival of patients with CC, demonstrating their potential as novel prognostic markers.

The results of cell-cell interaction analysis revealed active interactions between epithelial cell-*CXCL13*^*+*^ CD8^+^ TRM and epithelial cell-*PLAC8*^*+*^ CD8^+^ TRM in the TME of CC. The ligand-receptor pairs involved in these interactions included MHC class I molecules-related pairs (*HLA-A/B/C/E/F* - *CD8A/CD8B*) and T cell proliferation and activation-related pairs (*MIF*-(*CD74*+*CD44*) and *MDK*-*NCL*). Tumor cells can express various MHC class I molecules (e.g., HLA-A, HLA-B, and HLA-C) on their surface, which present tumor-specific antigens to CD8^+^ T cells. In some cases, cancer cells can down-regulate MHC class I expression, thus evading recognition and killing by T cells^42,43^. Notably, we observed that the interaction between *HLA-E* and *NKG2A* (either alone or in a complex with *CD94*), which is associated with T cell inhibition and increased cancer growth, was enriched in *CXCL13*^*+*^ CD8^+^ TRMs interacting with epithelial cells at CC tissues. NKG2A and its heterodimeric CD94:NKG2A form are inhibitory receptors that interact with HLA-E on target cells. This interaction transmits inhibitory signals to NK cells and CD8^+^ T cells, dampening their cytotoxic activity^23,24^. It has been shown that CC-infiltrating CD8^+^ T cells exhibit higher expression of CD94:NKG2A than those present in the peripheral blood or normal cervical tissues. Moreover, kinetic co-culture experiments have shown that CC cells promote CD94:NKG2A expression on CD8^+^ T cells. The increase in CD94:NKG2A expression on CD8^+^ T cells markedly reduce their intracellular perforin levels^44^. Therefore, the use of NKG2A inhibitors, typically antibodies which block the HLA-E and NKG2A interaction, has emerged as a promising immunotherapeutic approach in CC.

Although our study has provided valuable insights into the role of TRMs in CC, it is important to acknowledge several limitations. First, the sample size in our study is relatively small, and larger-scale studies are required to validate and reinforce our findings. Second, the HPV infection status of a portion of the normal cervical tissues was unknown in our study. Therefore, it was impossible to directly compare the immunological differences between normal tissues in which the virus had been cleared or was not detectable, and those in which the tissue appeared morphologically normal but the virus had been detected. Third, further functional experiments are needed to validate the characteristics of key TRMs subsets identified in this study. Lastly, additional spatial information is needed to understand the true localization of TRMs in CC.

In summary, this study comprehensively evaluated the heterogeneity and gene expression profiles of TRMs in CC, as well as their potential functions and interactions in the TME of CC at a single-cell level. Our findings revealed that TRMs are highly immunoreactive, capable of infiltrating tumor tissues, and displaying distinct gene expression profiles. Moreover, they play a significant role in CC eradication, potentially by recruiting other immune cells and responding to viral stimuli. These findings significantly contributed to our knowledge of TRM-mediated immunotherapy in CC, underscoring the potential of TRMs as prognostic markers and therapeutic targets. Further studies exploring the functional mechanisms and dynamics of TRMs in the TME of CC are warranted to fully comprehend their roles in the anti-tumor/viral immune response and to develop targeted therapeutic strategies for CC.

## Methods

### Human samples and data collection

The scRNA-seq data of 38,572 T cells included in this study comprised new data of 20,976 (54.4%) T cells from 3 HPV^+^ CC samples and previously published data of 17,596 (45.6%) T cells from 8 HPV^+^ CC and 5 normal cervical samples^45,46^. This study was approved by the Ethics Committee of Shandong Cancer Hospital (SDTHEC201906009) and conducted in accordance with the guidelines of the Declaration of Helsinki. Written informed consent was obtained from all participants. A total of three patients (age range: 32–53 years) with CC, all of whom were diagnosed with HPV16-positive tumors, were newly enrolled in this study after obtaining their written informed consent. Two patients were diagnosed with stage IIIC and stage IB2 cervical adenocarcinoma, according to the Federation International of Gynecology and Obstetrics classification. The third patient was diagnosed with stage IB2 cervical squamous cell carcinoma (Supplementary Table 1). Fresh tissues were obtained from the primary tumors of these patients through surgery. The tissues were promptly placed in MACS Tissue Storage Solution (Miltenyi Biotec) while kept on ice. Subsequently, the samples were promptly transferred to the laboratory.

### Sample preparation

For each tumor specimen, a serious of digestion and sorting steps were performed. First, the samples were enzymatically digested using a medium containing collagenase IV (2 mg ml^−1^) and Dnase I (1 mg ml^−1^) (both from Sigma), and then incubated at 37 °C for a duration of 35 minutes. To stop the digestion process, phosphate-buffered saline (PBS), supplemented with 1% bovine serum albumin (BSA) (Absin), was added. The digestion mixture was filtered using a 70-µm cell filter (BD Biosciences) and centrifuged at 400 × *g* for 5 min. After carefully removing the supernatant, the resulting cell pellet was treated with RBC Lysis Buffer (BioLegend) and incubated at 4 °C for 5 min. Following another centrifugation step at 400 × *g* for 5 min, the supernatant was discarded, and the resulting cell pellet was resuspended in 100 μl sorting buffer (PBS containing 1% BSA). Mouse anti-human monoclonal antibodies specifically targeting CD45 (Clone 2D1; BD Biosciences) and CD3 (Clone OKT3; BD Biosciences) were used to label the cells. The cells were incubated with the antibodies for 15 min at 4 °C, followed by two washes and resuspension in 100 μl of sorting buffer. Subsequently, the cells were stained with 7-AAD Viability Staining Solution (eBioscience) and sorted on the BD FACSAria III cell sorter, gating on live T cells (7-AAD^−^CD45^+^CD3^+^), for subsequent single-cell library preparation and sequencing.

### scRNA‐seq and data preprocessing

The scRNA-seq data were generated using the 10× Genomics platform. The raw sequencing FASTQ files were aligned to the GRCh38 reference genome using Cell Ranger count, utilizing the STAR algorithm were aligned to generate a gene expression library. The gene expression data obtained from the count matrices generated by Cell Ranger were processed using Seurat (version 4.2.0)^47^. The newly generated data were integrated with high-quality single-cell data from our previous studies^45,46^. The data was analyzed using Seurat for each batch, and the DoubletFinder R package was utilized to identify doublets. After removing the doublets, the raw gene expression data were filtered by excluding cells with high mitochondrial content or low gene expression counts. For the remaining high-quality cells, the gene expression matrix was normalized using the NormalizeData function. Highly variable genes were then selected using the FindVariableFeatures function with a threshold of 2000 features, and data standardization was performed using the ScaleData function. Next, principal component analysis (PCA) was conducted on the highly variable genes to identify the most significant principal components. To mitigate batch effects between samples, the data were integrated using the “harmony” R package.

### Cell type annotation and T cell isolation

The FindClusters function was used to identify cell clusters and subclusters, and the t-Distributed Stochastic Neighbor Embedding (t-SNE) algorithm was employed for dimensionality reduction and cell visualization. The resulting cell clusters were annotated based on the expression of lineage-specific genes in different cell types. After quality control and filtering, scRNA-seq profiles were obtained for 38,572T cells (*CD2*^+^ and *CD3D*^+^) (Supplementary Fig. 1a, b). Based on the expression of the known TRMs marker gene *ITGAE* (encoding CD103)^10^, we classified T cells as TRMs or non-TRMs using normalized expression data. The threshold was *ITGAE* expression greater than 0.5. Additionally, we obtained the scRNA-seq data of PBMC from three healthy individuals from the Gene Expression Omnibus database (accession number GSE157007). We extracted T cell data for analysis through dimensionality reduction clustering analysis and compared the expression of *ITGAE* in TRMs, non-TRMs, and T cells in PBMC. The differential proportion analysis for cell subclusters was conducted using the Wilcoxon test.

### Differentially expressed genes and pathway enrichment analysis

DEGs were identified using the FindAllMarkers function in Seurat, with the Wilcoxon test and significance threshold at *p*-value < 0.05 and an absolute log2 fold change >0.25. To investigate the functional relevance and activity of the gene sets, GO term analysis and GSEA were carried out using the ClusterProfiler package in R, focusing on the DEGs^48^. In addition, the activity levels of signaling pathways were compared between different cell types by scoring gene signatures using the Ucell R package^49^.

### Analysis of data from The Cancer Genome Atlas

Bulk RNA-seq data were used alongside curated clinical data from TCGA database. The top 30 DEGs in TRMs were selected to define the T cell population characteristics. Subsequently, Gene Set Variation Analysis (GSVA) was conducted to evaluate the relative abundance of these cell signatures in TCGA database^50^. The survcutpoint function from the survminer R package was employed to determine the optimal cutoff value. Using this cutoff, the Patients with CC following radiotherapy were categorized into high- or low-expression groups based on their gene expression profiles. The survival package as used to perform the log-rank test and the Kaplan–Meier survival curves were plotted by the ggsurvplot function.

### Predicting putative interactions between cell types

The CellChat R package was utilized to infer putative interactions between different cell types by analyzing receptor-ligand interactions within the scRNA-seq dataset^51^. We first normalized the Seurat object using the NormalizeData function of the Seurat package, which scales gene expression measurements to obtain comparable data from different cells. After normalization, data were stratified by tumor and normal tissues to explore potential cell interactions within the tumor ecosystem. Preprocessing functions, such as identifying overexpressed genes and interactions, as well as data projection, were executed using default parameters. For the primary analyses, core functions, including computing communication probabilities, computing pathway-specific communication probabilities, and aggregating the network, were executed separately for tumor-free and tumor tissue datasets. The results were merged using the mergeCellChat function. The results obtained were merged using the mergeCellChat function. To visually represent the communication probabilities mediated by ligand-receptor pairs between specific cell groups, the netVisual_bubble function was utilized. Additionally, netVisual_individual was used to depict all the important interactions between a given signal and the associated signaling genes.

### Multiplex immunofluorescent labeling

To verify the difference in the infiltration degree of TRMs and non-TRMs between CC tissues and normal cervical tissues, we obtained 20 FFPE samples of CC and 20 normal cervical tissue samples. The following markers were used for immunofluorescent labeling: CD103, CD3, and 4,6-diamidino-2-phenylindole (DAPI) for nuclear staining. Immunofluorescence kit designed for multiplex staining was used. FFPE tissue sections of CC and normal cervical tissues were sequentially incubated with the above primary antibodies. The tissues were subsequently incubated with horseradish peroxidase-conjugated secondary antibodies, and signal amplification was achieved using tyramide signal amplification (TSA). Each step was followed by heating in a microwave to facilitate antibody stripping and prepare the sections for subsequent labeling. After all target antigens were labeled, nuclear counterstaining was performed using DAPI. Stained slides were scanned with a Mantra System (PerkinElmer), capturing fluorescence spectra across 420–720 nm at 20-nm intervals, under consistent exposure times. The resulting spectral images were processed using the InForm image analysis software (PerkinElmer). Use Visiopharm Intelligent Full Line AI Digital Pathology Quantitative Analysis Software to perform target cell counting on the entire resulting spectral images.

To identify epithelial cells, *CXCL13*^+^ CD8^+^ TRMs, and their spatial relationship within the TME of CC, a multiplex immunofluorescent labeling technique was utilized. This approach employed markers including Pan-CK to detect epithelial cells, CXCL13, CD103, and CD8 to identify *CXCL13*^+^ CD8^+^ TRMs, and DAPI for nuclear staining. Immunofluorescence kit designed for multiplex staining was used. FFPE tissue sections of CC were sequentially incubated with the above primary antibodies followed by incubation with horseradish peroxidase-conjugated secondary antibodies. Signal amplification was achieved using TSA. Each TSA step was followed by heating in a microwave to facilitate antibody stripping and prepare the sections for subsequent labeling. After all target antigens were labeled, nuclear counterstaining was performed using DAPI. The stained slides were scanned using a Mantra System (PerkinElmer), capturing fluorescence spectra across 420–720 nm at 20-nm intervals, under consistent exposure times. The resulting spectral images were processed using the InForm image analysis software (PerkinElmer).

### Supplementary information

Supplementary Data

## Data Availability

Processed scRNA-seq data were uploaded to the National Genomics Data Center, which are publicly accessible at https://ngdc.cncb.ac.cn/omix (OMIX006446-01).

## Code availability

The data analysis R script for this study is publicly accessible at https://github.com/WFhoo/TRM. Conducted using R version 4.1.3, the script includes detailed documentation of the specific parameters and variables utilized.
