# Supplementary material for: Cellular heterogeneity and key subsets of tissue-resident memory T cells in cervical cancer
Source: NPJ Precis Oncol. 2024 Jul 16;8:145. doi: 10.1038/s41698-024-00637-3 (PMC11252146; doi:10.1038/s41698-024-00637-3)
Supplement: Supplementary file 1 — Supplementary Data [file 41698_2024_637_MOESM1_ESM.pdf]

Cellular heterogeneity and key subsets of tissue-resident memory T cells in cervical cancer

Authors:

Fuhao Wang[1], Shengqin Yue[2], Qingyu Huang[3], Tianyu Lei[2], Xiaohui Li[3], Cong Wang[4], Jinbo Yue[3*], Chao Liu[1*]*

The PDF file includes:

Supplementary Figure 1 to 6, Supplementary Table 1.

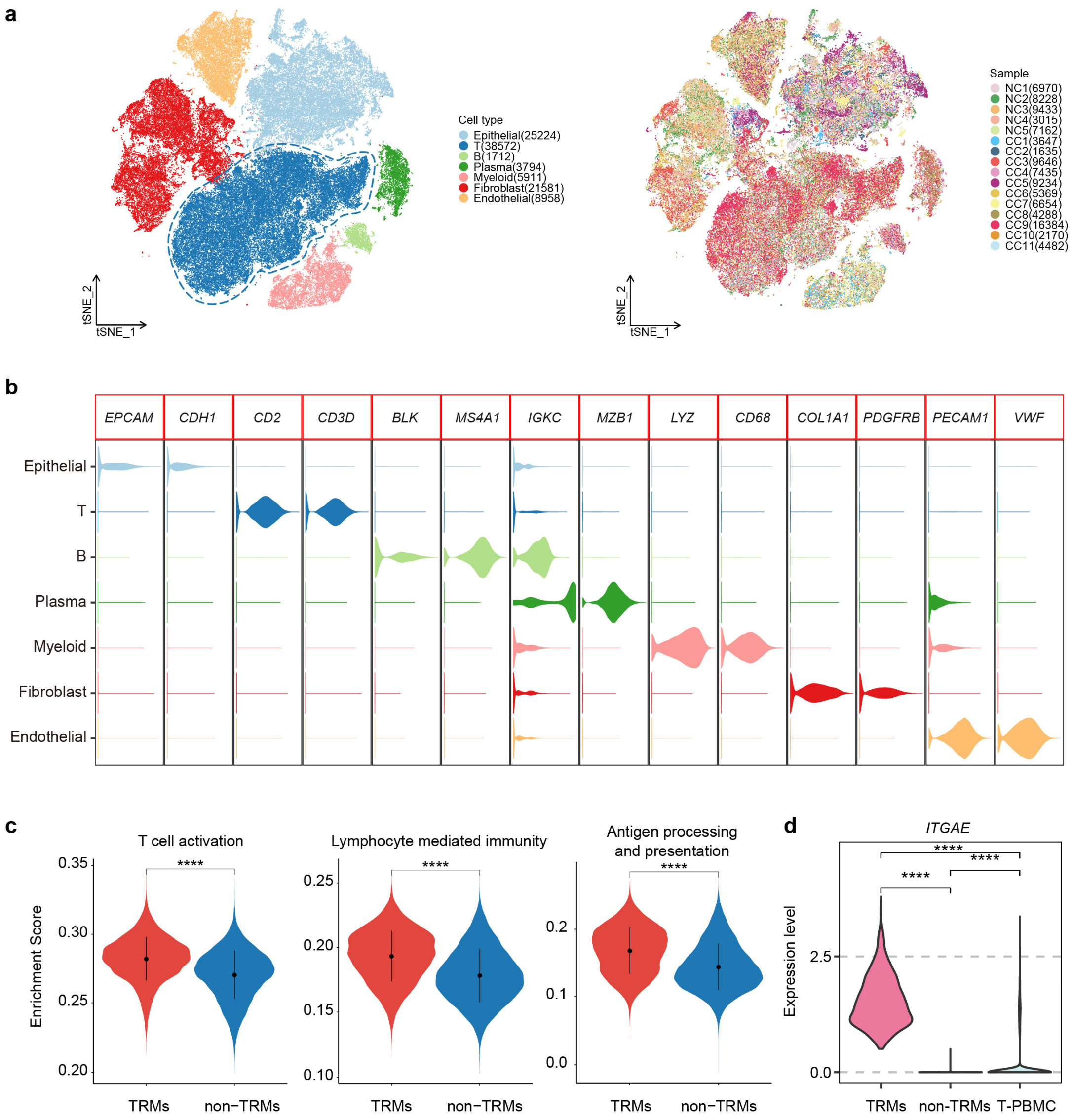

Supplementary Figure 1. Identification of the main cell types from scRNA-seq data and the differences in pathway activity between TRMs and non-TRMs. (a) t-Distributed Stochastic Neighbor Embedding (tSNE) plot visualization of all the identified cell types within the scRNA-seq dataset, color-coded by cell type (left) or sample origin (right). **(b)** Violin plots showing the expression of known marker genes, used to identify the different cell types. **(c)** Violin plots showing differences in pathway activity between TRMs (red) and non-TRMs (blue). *, $p<0.05$; **, $p<0.01$; ***, $p<0.001$; ****, $p<0.0001$ (two-sided Wilcoxon test). **(d)** Violin plot showing the expression of *ITGAE* in TRMs, non-TRMs, and T cells in peripheral blood mononuclear cells (T-PBMC); ****, $p<0.0001$.

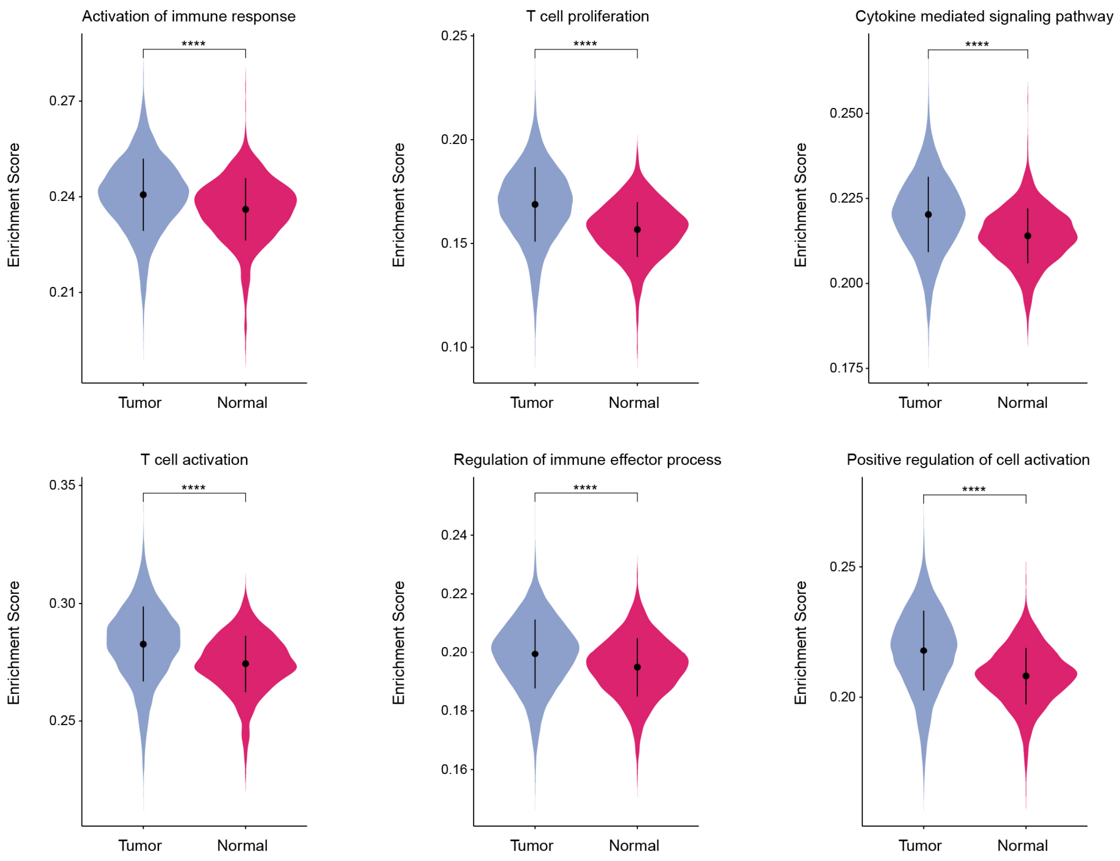

Supplementary Figure 2. Differences in the pathway activities of TRMs derived from cervical cancer tissues or normal cervical samples. Violin plots depicting differences in the activities of the indicated pathways between TRMs from cervical cancer tissues and those from normal cervical tissues. *, p<0.05; **, p<0.01; ***, p<0.001; ****, p<0.0001 (two-sided Wilcoxon test).

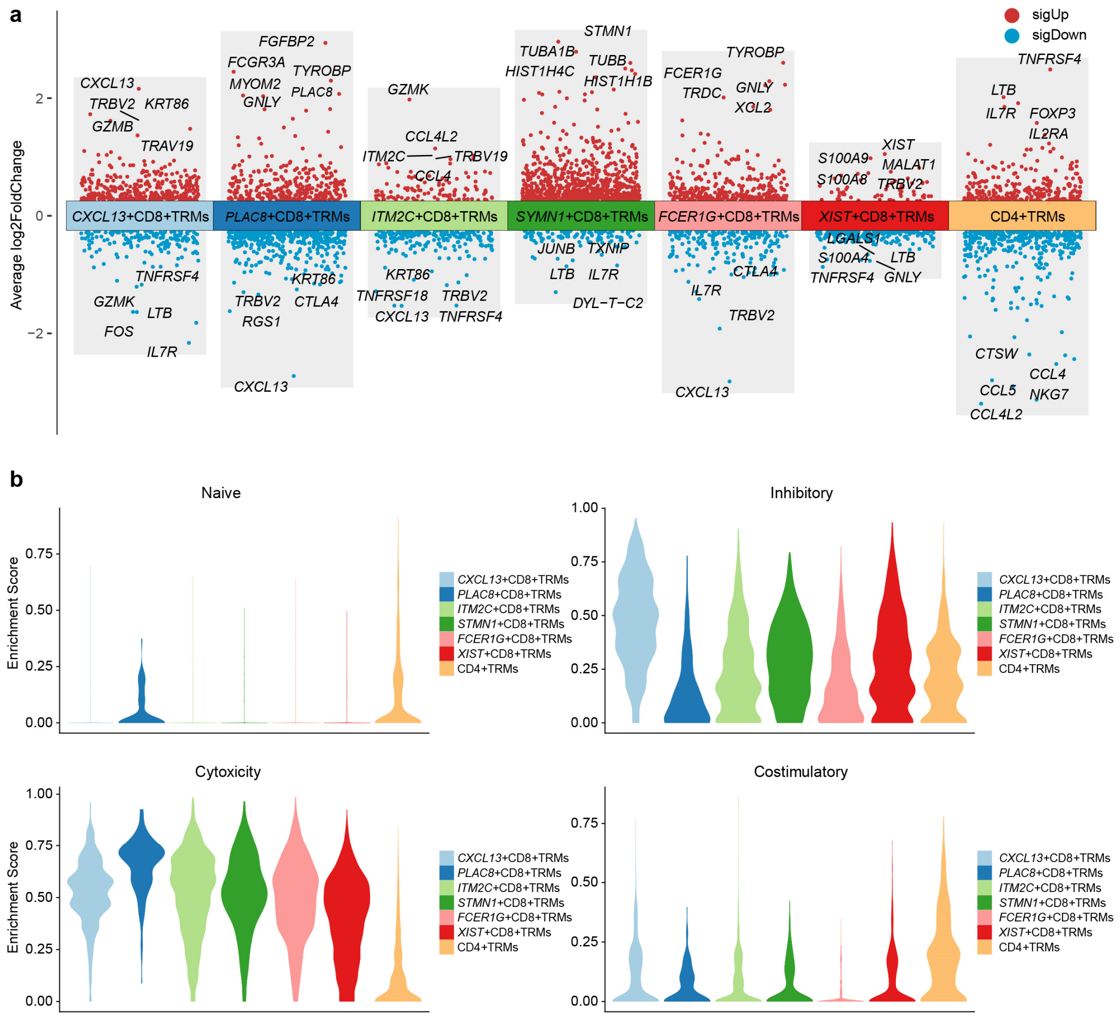

Supplementary Figure 3. Transcriptomic features of the seven TRM subclusters. (a) Volcano plots showing the relative expression levels of differentially expressed genes in the seven TRM subclusters. **(b)** Violin plots showing the enrichment scores of functional signature genes in the seven TRM subclusters, color-coded by cell type.

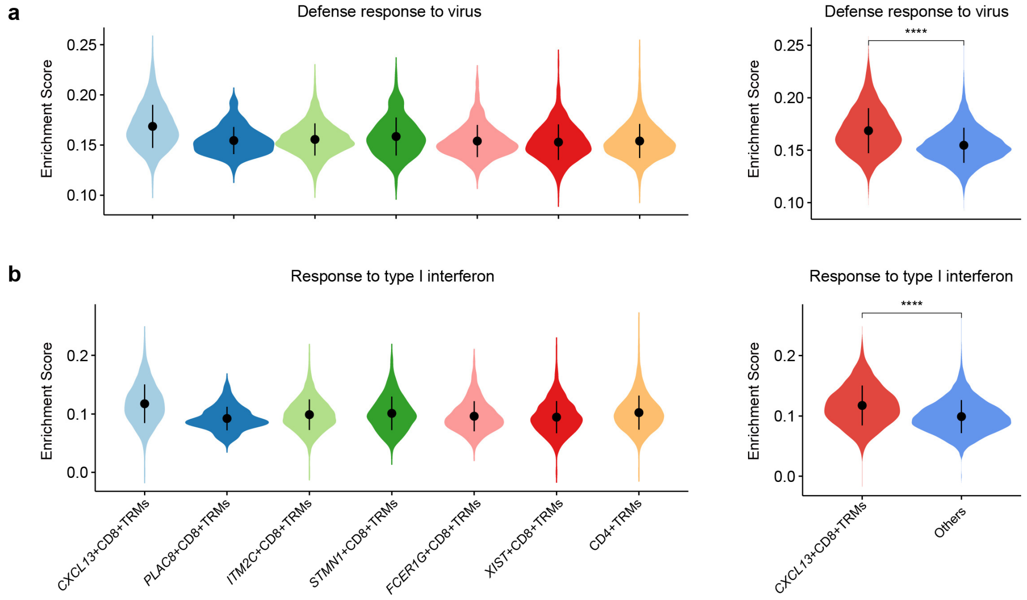

Supplementary Figure 4. Differences in the indicated pathway activities between *CXCL13*$^{+}$ CD8^{+} TRMs and the other TRM subclusters. Violin plots showing the indicated pathway activities of the seven TRM subclusters (left) and differences between the pathway activities of *CXCL13*$^{+}$ CD8^{+} TRMs and those of the other TRM subclusters (right), color-coded by cell type. *, *p*<0.05; **, *p*<0.01; ***, *p*<0.001; ****, *p*<0.0001 (two-sided Wilcoxon test).

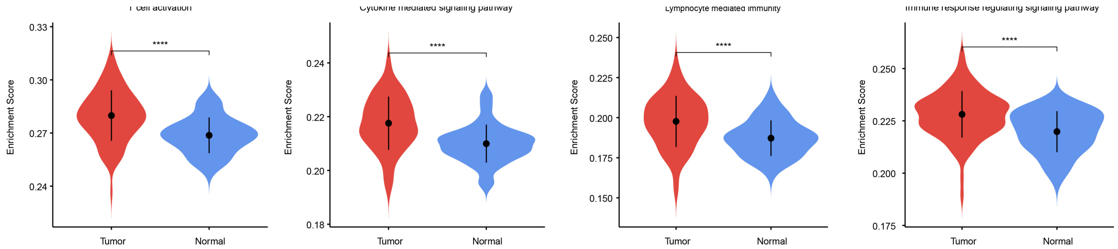

Supplementary Figure 5. Differences in the indicated pathway activities between *PLAC8*$^{+}$ CD8^{+} TRMs from cervical cancer tissues and those from normal cervical tissues. Violin plots showing the indicated pathway activities of *PLAC8*$^{+}$ CD8^{+} TRMs in cervical cancer (red) and normal cervical samples (blue). *, *p*<0.05; **, *p*<0.01; ***, *p*<0.001; ****, *p*<0.0001 (two-sided Wilcoxon test).

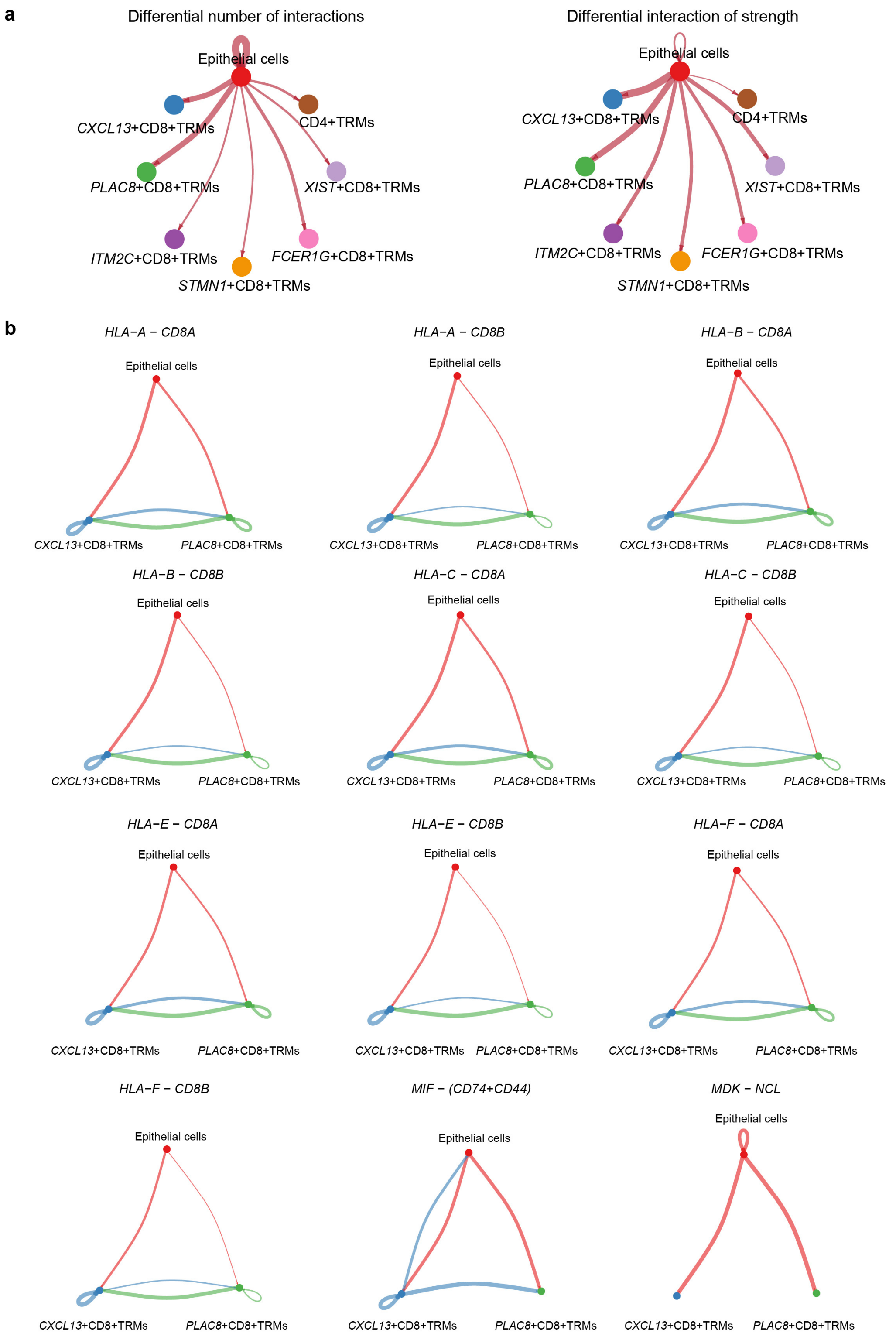

Supplementary Figure 6. Crosstalk between epithelial cells and TRM subclusters. (a) Circle plots displaying the number and strength of interactions between epithelial cells and TRM subclusters in cervical cancer tissues, respectively. **(b)** Circle plots showing the indicated ligand-receptor pairs involved in the interaction between epithelial cells and either *CXCL13*$^{+}$ CD8^{+} TRMs, or *PLAC8*$^{+}$ CD8^{+} TRMs in cervical cancer tissues; color-coded by cell type.

Supplementary Table 1. Patients and samples collection.

Samples	Pathology	FIGO stage (2018)	HPV infection
CC9	Squamous cell carcinoma	IB2	16+
CC10	Adenocarcinoma	IIIC	16+
CC11	Adenocarcinoma	IB2	16+
CC1	Squamous cell carcinoma	IIIB	16+
CC2	Squamous cell carcinoma	IIIB	16+
CC3	Squamous cell carcinoma	IIB	16+
CC4	Squamous cell carcinoma	IB2	16+
CC5	Squamous cell carcinoma	IB2	16+
CC6	Squamous cell carcinoma	IIIC	16+
CC7	Squamous cell carcinoma	IB2	16+
CC8	Squamous cell carcinoma	IIIC	16+
NC1	/	/	/
NC2	/	/	/
NC3	/	/	/
NC4	/	/	18+
NC5	/	/	18+

Note: NC1, NC2, NC3 samples obtained from postoperative cervical tissues of patients with endometrial cancer, where the HPV infection status is unknown. CC, cervical cancer; NC, normal cervical tissues.
